# Back Numbers Wanted

**Published:** 1891-02

**Authors:** 


### WANTED BACK NUMBERS.

We will pay twenty-five cents each for a few copies in good condition of No. 2, Vol. IX, February, 1890.—EDITOR.

